# VBlock: A Blockchain-Based Tamper-Proofing Data Protection Model for Internet of Vehicle Networks

**DOI:** 10.3390/s22208083

**Published:** 2022-10-21

**Authors:** Collins Sey, Hang Lei, Weizhong Qian, Xiaoyu Li, Linda Delali Fiasam, Seth Larweh Kodjiku, Isaac Adjei-Mensah, Isaac Osei Agyemang

**Affiliations:** 1 School of Information and Software Engineering, University of Electronic Science and Technology of China, Chengdu 610054, China; 2 School of Computer and Information Engineering, Zhejiang Gongshang University, Hangzhou 310018, China; 3 School of Information and Communication Engineering, University of Electronic Science and Technology of China, Chengdu 611731, China

**Keywords:** blockchain, Internet of Vehicles (IoV), smart city, tamper-proof, Internet of Things (IoT), collusion resistance, key revocation

## Abstract

The rapid advancement of the Internet of Vehicles (IoV) has led to a massive growth in data received from IoV networks. The cloud storage has been a timely service that provides a vast range of data storage for IoV networks. However, existing data storage and access models used to manage and protect data in IoV networks have proven to be insufficient. They are centralized and usually accompanied by a lack of trust, transparency, security, immutability, and provenance. In this paper, we propose VBlock, a blockchain-based system that addresses the issues of illegal modification of outsourced vehicular data for smart city management and improvement. We introduce a novel collusion-resistant model for outsourcing data to cloud storage that ensures the network remains tamper-proof, has good data provenance and auditing, and solves the centralized problems prone to the single point of failure. We introduced a key revocation mechanism to secure the network from malicious nodes. We formally define the system model of VBlock in the setting of a consortium blockchain. Our simulation results and security analysis show that the proposed model provides a strong security guarantee with high efficiency and is practicable in the IoV environment.

## 1. Introduction

The smart transportation sector is one of the major sectors that has been transformed by the Internet of Things (IoT) concept with the inception of the Internet of Vehicles (IoV) [1]. The IoV network is a network that consists of vehicles with IoT-enabled devices deployed in them, interconnected to provide innovative services to form the smart city [2]. The IoT-enabled devices coordinate to form a network which integrates information. The information includes the location of vehicles, speed, and vehicle routes. This creates a trend of massive data collection, of which the cloud computing techniques provide a perfect solution for the vehicles to outsource unlimited storage resources for smart city management.

The field continues to attract huge research attention, as vast data are projected to emanate from the IoV network. In vehicular networks, data exchanges are needed to improve the management of smart cities. This creates significant importance and merits for the network. Although there are significant advantages, there are issues and challenges associated with the security and privacy of the data being exchanged [3,4,5]. The safety and efficiency of smart city management rely on valid data from the IoV network. Valid data imply data free from forgery and illegal modification. Data integrity should be maintained, for the usage of data by the various systems and services. Systems such as Intelligent Transport System (ITS), Traffic Management System (TMS), safety services departments (such as fire service, and ambulance), and forensic services [6] (crime investigation) heavily rely on valid data from the IoV network for their works. Invalid data from the IoV network will lead to serious problems. The 2021 Global Automotive Cybersecurity Report released by Upstream Security points out an increase of 75% in servers targeted for attacks in 2020 [7]. For this reason, IoV network data must meet security requirements such as authentication, integrity, privacy preservation, and data provenance described in [8]. To secure the data emanating from the IoV network, some authentication schemes and models have been proposed [9], such as identity-based cryptography (IDC), public key infrastructure (PKI), certificateless cryptography (CLC) [10], log management scheme [11], and proxy re-encryption [12], which focus on different security requirements. 

**Figure 1:**
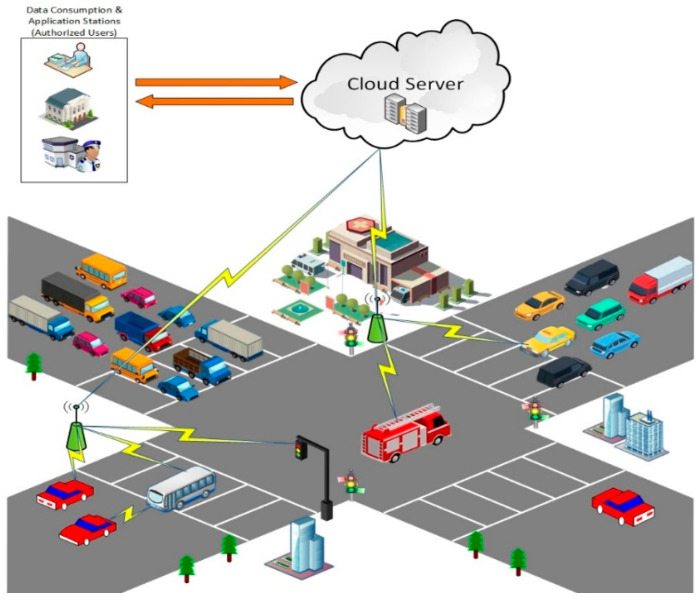
A typical scenario of IoV network.

Blockchain technology has shown great potential recently, and thus attracted interest from many researchers, computer scientists, engineers, etc., who have therefore proposed its application to diverse disciplines, which include intelligent transport systems [13,14]. It has shown great potential to provide a substantial number of innovative solutions to a majority of IoV application scenarios. The technology has the potential to make intelligent transport systems more secure, autonomous, distributed, and safe. Integrating blockchain into IoV not only improves security, privacy, and trust but also enhances system performance and automation. As we seek to ensure the integrity of data that smart city systems rely on, blockchain has proven to be crucial in realizing this goal. Blockchain technology represents an immutable ledger of transactions. By this, accountability and auditing can be achieved without relying on a single point of trust. In this work, we ensure tamper-proofing, data provenance, and auditing of IoV data in the cloud server scenarios, which prevent illegal modification of data even if the data generator colludes with the cloud server. Current security measures implemented in the IoV systems are centralized. To ensure tamper-proof outsourced IoV data, existing models utilize authentication mechanisms to authenticate the IoV nodes that are validated by these centralized authorities, such as the cloud server. In these models, a strong presumption exists that the IoV node will not collude with the cloud server to modify or tamper with the outsourced IoV data. If the IoV node or the user incentivizes the cloud server to tamper with the outsourced IoV data generated by itself, it is difficult to detect. Since the cloud server is viewed as a rational entity, it is feasible for a malicious node/user to compromise it, provided the cloud server is given the necessary incentives. Figure 1 shows a typical scenario of IoV network.

In this work, we leverage blockchains to address the issue of illegal modification of outsourced IoV data, even by the data generators themselves, in a model called VBlock. The main contributions of our work are summarized below:

- We design a secure model called VBlock for outsourcing data to the cloud by IoV nodes which ensures tamper-proofing, data provenance, and auditing. We utilize blockchain techniques to decentralize all data storing entities in the IoV network by integrating activities as transactions on the blockchain. The cloud server in our model only accepts the IoV data generated by the IoV node, provided the IoV node has a valid warrant, and a corresponding transaction is recorded on the blockchain for the data being outsourced. We describe this as warrant-based data outsourcing.
- We introduce a certificate/key revocation mechanism to ensure that all nodes communicating in the IoV environment are legitimate and have not been compromised by malicious activities.
- We conducted a series of experiments to evaluate the validity, security, and performance efficiency of our proposed model, and the results shows that our model is practical and efficient in the IoV scenario.

The rest of this paper is organized as follows. We summarize the related works in Section 2. In Section 3, we formulate the problem statement. We discuss the preliminaries and core technologies used for this research in Section 4. Section 5 details the proposed model. Section 6 and Section 7 present the security analyses and performance evaluation. respectively. We present our concluding remarks and future work in Section 8. 

## 2. Related Works

Security and privacy protection in IoV systems continue to be the focus for researchers due to standardization differences in device production, centralized storage, and access models. Significant research proposals have been presented which enable secure data access model in the usual client–server architectures. Other IoT providers use techniques that constitute proprietary authorization. In such situations, they serve as centralized authorizing entities. Nonetheless, a major challenge associated with centralized IoT data management is scalability and trust issues. The security of such systems is solely based on trust in the central server. A compromise of the central server leaves the entire system void. Ensuring data security of IoV data for safety and efficient management of the smart city is very crucial. This has attracted significant research focus on incorporating blockchain technology into IoV systems to decentralize data security and privacy. 

Vallent et al. [15] proposed a certificateless signature scheme based on elliptic curve cryptography that preserves privacy in VANET. Their scheme sorts out the KGC escrow problem and uses time and pseudo-identity to validate communicating vehicles while excluding bilinear pairings. Liu et al. [16] presented a scheme for defense against malicious nodes in VANET based on blockchain. They constructed two types of blockchains that can identify malicious nodes and forged messages through reputation, time and distance.Ma et al. [17] presented a blockchain-based secure, privacy-preserving, and decentralized IoV architecture. Their work introduced a hierarchical data-sharing framework with two types of sub-blockchain for flexible access control. Their architecture consists of vehicles, actuators and sensors, roadside units (RSUs), and cloud computing nodes. They designed a lightweight consensus algorithm based on a reputation that uses a multi-weight reputation technique. Kang et al. [18] utilized consortium blockchain and smart contract to secure data storage and sharing in vehicular edge computing networks. The authors used the technologies to ensure that the data could not be shared without the necessary authorization, employing a reputation-based data-sharing scheme to help vehicles achieve high-quality data sharing. Their reputation scheme improves malicious vehicle detection compared to traditional reputation schemes. Javaid et al. [19] proposed DrivMan, a blockchain-based solution for Internet of Vehicles, which ensures trust management, data provenance, and privacy via smart contract, public key infrastructure (PKI), and physically unclonable function (PUF). PUF helped to provide a unique crypto fingerprint to every vehicle. They used a certificate authority (CA) to register vehicles, and also revoke their registration certificate when necessary. Shi et al. [20] presented a multimedia data-sharing model based on blockchain and cryptographic primitives for vehicular social networks. They used cryptographic primitives to conceal the identity of users, vehicles, and RSUs. On the other hand, blockchain was used to ensure the immutability of reliable data sources and prevent attackers from forging or tampering with multimedia data. Ali et al. [21] proposed a blockchain-based certificateless public key signature with bilinear pairing for VANETs that enable conditional privacy-preserving authentication. They used blockchain to efficiently implement transparency of pseudo-entities prior to signature verification. Su et al. [22] presented a privacy protection system based on blockchain for the Internet of Vehicles. They designed a two-way authentication and key agreement algorithm, which eliminates the central point of failure problem associated with the traditional IoV system. However, their model makes use of a proof-of-work consensus algorithm that consumes more computing resources. Malik et al. [23] presented a novel method of mutual authentication and privacy protection for nodes in a vehicular ad hoc network (VANET). They used a private blockchain with an access control layer on the shared ledger and introduced a certificate revocation mechanism to secure the VANET from malicious nodes. Ratep et al. [24] presented a blockchain-based decentralized IoT solution for vehicles communication (DISV) to secure vehicle-to-everything (V2X) communication in a decentralized cloud computing environment. They used a three-layer approach to explore communication via blockchain on an Ethereum blockchain. However, the base guest price in Ethereum deployment will limit their scalability. Recent literature has attracted attention in ensuring the integrity of outsourced data [25,26,27]. These schemes ensure that data outsourcing to the cloud server is done by the trusted data generators themselves. However, if a malicious node (generator) tampers with the outsourced data that it generated already by incentivizing the cloud server, it is quite hard to detect. Furthermore, schemes rely on centralized authorities to achieve trust in data management. A comprehensive survey of blockchain for IoV networks has been discussed in [28,29,30].

## 3. Problem Statement

### 3.1. Cloud-Based IoV Network

There are basically three different entities in the IoV system: the IoV node (the vehicle), the cloud server, and the authorized data users (police, government/research institutions, intelligent systems). The procedure for outsourcing IoV data in the IoV system is illustrated as follows.

First, the IoV node is registered onto the IoV system with a unique ID provided by the manufacturer. The unique identity of the vehicle is strictly reviewed in order to ensure the legitimacy of the nodes in the system. Authorization keys are generated for access to the cloud server services. The same process is done for authorized data users. The IoV node encrypts the data (GPS Location, Travel Time Index) generated or collected and outsources them to the cloud server. The cloud server authenticates the node by verifying the validity of the key being used. The authorized data users retrieve the outsourced data for further analysis or usage in other smart city systems such as the intelligent transport system and traffic management system. The efficiency of these systems greatly depends on the data accessed from the cloud storage server, and any illegal modification of this data will cause serious problems. 

From the description above, the data is generated, encrypted, and uploaded by the data owner. This makes it very challenging to ensure illegal modification of the already outsourced data by the data owner does not take place. In this situation, security and efficiency challenges are introduced. 

The general overall safety and efficiency of smart city designs make use of the data collected and outsourced to the cloud. In the case of the Smart Traffic System, the IoV devices deployed on the vehicles constantly broadcast their location information to the cloud to enable the Smart Traffic System to make accurate analysis and controls with the data received from these vehicles. Attackers can manipulate the data collected and stored by the cloud server to create a misrepresentation of the actual location data in the cloud server. In a case of criminal investigation, gathering evidence is an important aspect of an active police investigation [6]. Data such as GPS tracking information can serve as evidence that helps in crime investigation. Typically, an attacker may forge, modify or delete outsourced IoV data to conceal such malicious activity or criminal acts that have been recorded by the vehicle and could aid the security services (police) in criminal investigation.

### 3.2. Threat Model

Here, we analyze the potential threats in the IoV system. In this threat model, we consider threats from two different perspectives: the external threat and the internal threat.

#### 3.2.1. External Threat

External threats target systems by impersonating an IoV node to outsource data to the cloud. In the existing system, IoV nodes encrypt data with their private key information and then outsource the data to the cloud. External threats may be able to acquire the master secret key of other IoV nodes. However, they may not be able to replace public keys. One-way chosen ciphertext attack OW-CCA security is defined by this type of threat.

#### 3.2.2. Internal Threat

- Rational Storage server. We follow the same assumptions made in [26,31], that the cloud server is a rational entity. This simply implies that the server will only deviate from the expected protocol if such a strategy increases benefits in the system.
- Semi-trusted IoV node. Users of the IoV nodes can all be classified as semi-trusted nodes. By this, we mean the nodes will operate normally during everyday scenarios. However, the user of the IoV node (attacker) may perform the following attacks.
  1. The attacker may collude with the cloud server, outsource forged IoV data to conceal evidence needed for criminal investigation or cause data integrity problems that affect smart city management.
  2. The attacker may violate the integrity of IoV data outsourced. The attacker may collude with the cloud server to illegally modify or delete portions of outsourced data from other nodes on the cloud server.

### 3.3. Design Goals

In this paper, we focus on the security of outsourced IoV network data to the cloud server in vehicular networks, where the following challenges exist:

1. How collusion between malicious deployed IoV node (attackers) and the cloud server can be avoided in the IoV network. A strong assumption exists in the current cloud-assisted data storage of IoV networks, that the cloud server will not collude with the IoV node or the user to modify outsourced IoV network data.
2. How to securely timestamp the IoV data before outsourcing to verify the legitimacy of outsourced data and avoid replay attacks by malicious nodes. It is very important to accurately maintain and securely timestamp the data from the IoV network.
3. How to securely authenticate the IoV nodes. Current cloud-assisted IoV networks utilize the traditional PKI schemes such as central authority (CA) or key generation center (KGC), which are prone to a single point of failure when data are tampered with in the CA or KGC.
4. How to ensure trust in outsourced data in a cloud-assisted IoV system by eliminating the single point of trust in the cloud server. The current server-aided model is limited to a single point of trust in the outsourced cloud server where the security of data no longer holds when the server is compromised.

## 4. Preliminaries

### 4.1. Notations, Conventions, and Basic Theory

Notation: *We denote* x∥y *as the concatenation of two bits strings*  x *and* y*. We use* $Enc_{CL}$*() to denote certificateless public key encryption*.

Bilinear Maps: 

Let $G_{1}$ and $G_{2}$ be two cyclic additive and multiplicative groups, respectively, with the same prime order q. P is the generator of $G_{1}$. A bilinear map $e:G_{1}\times G_{1}\to G_{2}$ should satisfy the following properties.

- Bilinearity: $e\left(aP,bP\right)=e{\left(P,P\right)}^{ab}=e\left(P,abP\right)=e\left(abP,P\right)$ for all $a,\;b\;\in z_{q}^{*}$
- Non-degeneracy: For $P,Q\in G_{1}$, $e\left(P,Q\right)\neq 1$ where 1 is the identity element in $G_{2}$.
- Computability: There exists an efficient computable algorithm to compute $e\left(P,Q\right)$ for $P,Q\in G_{1}$.

### 4.2. Cryptographic Keys

Cryptographic keys employed in this model are used to guarantee the security of the model used and the process. We encrypt data generated to protect its confidentiality for communication between devices via untrusted channels. These keys provide the necessary assurance of high-level data security for our model. The processes of outsourcing data to and sending and receiving query responses make use of cryptographic primitives to achieve secure communication, free from unwanted/malicious activities such as eavesdropping. Cryptographic keys are also employed at the data consumption level to ensure the authenticity of users requesting access to the outsourced data. We adopt the certificateless public key cryptography system, where the process of generating a key is divided into two parts. The KGC provides the first part, which is the partial private key, and the user combines it with his/her secret value to generate the full private key. Usually, the Key Generation Center (KGC) generates keys for usage in cryptosystems. However, in a case of a compromised KGC, the whole system breaks down. To solve this, Certificateless cryptography was introduced. All cryptographic operations are done by the user using the full private key, which consists of the user’s secret value and the partial private key generated by the KGC. Here, the KGC cannot have access to the user’s data because it has only partial access to the private key.

### 4.3. Blockchain

**Figure 2:**
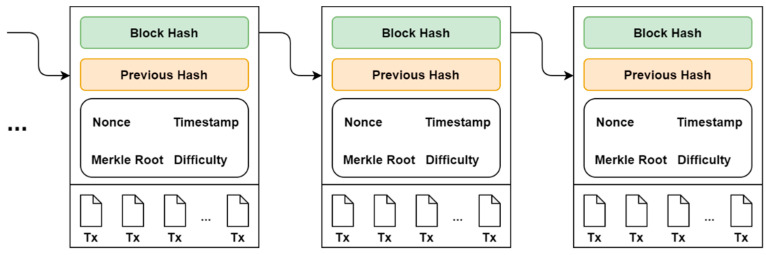
A simplified Blockchain Structure.

The blockchain is a classical distributed database that shares transactional records that are linked together across a peer-to-peer network. Records are shared in such a way that all participants in the network hold the same copy of the database. No central authority is needed in this network, and no single node or participant can control the whole peer-to-peer network. Blockchain technology has recently attracted interest from many computer scientists and domain experts in various industries and academia. Cryptocurrency (such as Bitcoin [32], Ethereum [33], and Zcash [34]) has been a great application of this technology. Lately, the financial field has received major backing with blockchain technology. Nevertheless, it has been very useful in many other non-financial fields such as identity-based PKI [35], supply chain, crowdsourcing [36], decentralized proof of document existence, decentralized IoT [37], decentralized storage [38], and electronic health systems [39]. Blocks are linked together in series and are added by consensus algorithm among the participating nodes. Blocks are mathematically verified using cryptography to ensure that they follow in order from the previous block. The cryptographic scheme used in the consensus protocol makes the network immutable and tamper-resistant. Figure 2 shows a simplified blockchain structure.

### 4.4. Hyperledger Fabric Blockchain

**Figure 3:**
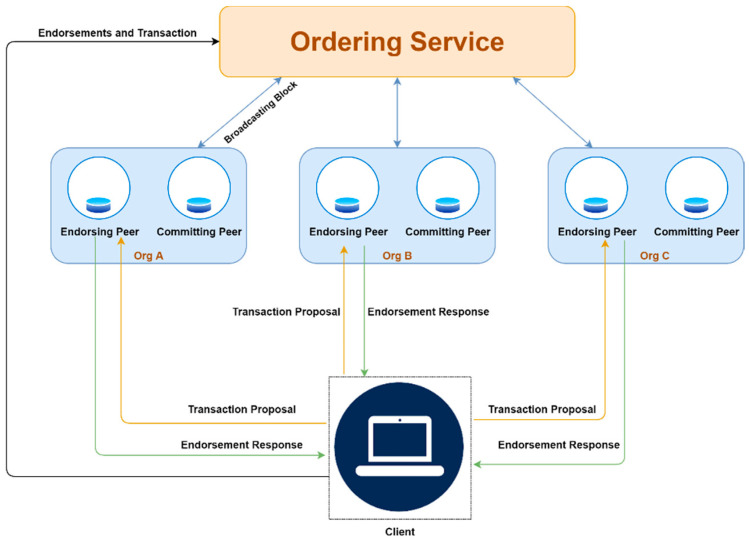
Fabric blockchain network operated by three organizations.

Fabric is the most common open-source permissioned blockchain platform project developed by the Linux Foundation. The project, which consists of other platforms, includes Hyperledger Sawtooth, Hyperledger Iroha, Hyperledger Burrow, and Hyperledger Indy. Three types of nodes basically form the Hyperledger Fabric network. These nodes are peers, customers, and clients. A membership service provider (MSP) that is owned by participating organizations is used to identify nodes on the network. Fabric utilizes a three-phase protocol, execute–order–validate, to complete transactions. Figure 3 shows an example of a Fabric blockchain network operated by three organizations. 

Clients send transaction proposals to the endorsing peers specified by the endorsement policy. The endorsing peers validate the transaction proposal, execute the chaincode (smart contract) and send a response to the client. The client waits to get enough responses specified by endorsing policy and then sends the responses as a transaction to the ordering service. The transaction contains the set of endorsements, the metadata of the transaction, the transaction payload, and the channel ID. The ordering service is not permitted to check the content of the transactions. It uses the plugged consensus mechanism to order transactions into blocks and broadcast them to the committing peers using the gossip protocol. Each peer independently validates the transactions and commits the blocks to their locally stored ledger state.

## 5. Architecture of VBlock

**Figure 4:**
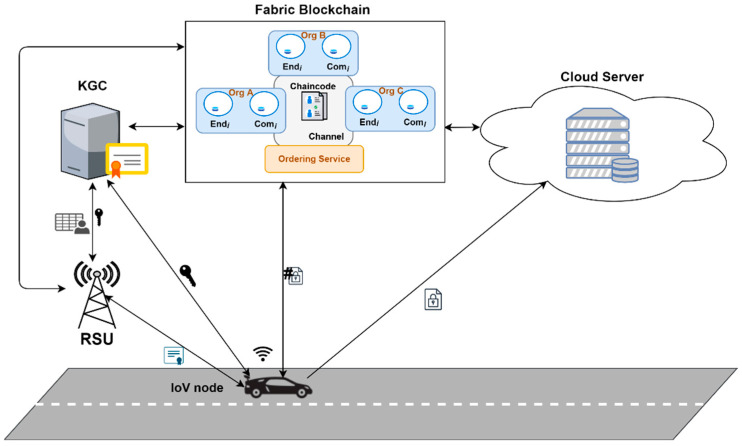
VBlock architecture with key components.

We propose VBlock, a model based on blockchain technology in smart city vehicular networks that allows for the development of a decentralized network of large-scale vehicular data security more effectively and efficiently. With this architecture, we aim to provide IoV data security via blockchain and also address the challenges associated with deploying blockchain to IoV networks. In selecting the type of blockchain suitable for our model, we consider the following factors: the number of transactions per second, confirmation time, security and access control, participation cost, and fault tolerance on the network. The IoV network requires a high-performing blockchain platform that supports a high number of transactions per second with a low confirmation time. The use case designed in our model requires such a type of blockchain and hence makes the consortium blockchain best suited for our model. We model VBlock in a consortium blockchain where access to the ledger is confined to previously validated and registered members via only a Hyperledger Fabric blockchain to increase the security and privacy of data. Figure 4 shows the architectural overview with the key components of our model.

### 5.1. Choice of Blockchain Platform

Blockchain systems rely on consensus mechanisms to keep the network running. Ethereum and Hyperledger being the most popular blockchain platforms, are both great platforms; however, they target different use cases and have different consensus mechanisms. Hyperledger Fabric is a permissioned blockchain and therefore ensures strict control over members of the network. Only preconfigured, authorized members can have access to the network. This ensures higher data privacy, security, and confidentiality as compared to the Ethereum blockchain, which is public, with transactions being transparent to any participants that join the network. Although Ethereum now introduces the PoS consensus mechanism, it still requires some base guest price to keep the network running as compared to Hyperledger, which does not require any. Hyperledger comparably has lower confirmation time and higher throughput. For this work, Ethereum did not fully meet the needed requirements of our IoV network design, which requires higher transaction speed with higher data privacy, security, and confidentiality. Moreover, the cost-free deployment feature of Hyperledger makes it more suitable for the IoV network as compared to Ethereum, which requires the base guest price for every transaction on the network. For these reasons, we chose Hyperledger Fabric Blockchain.

### 5.2. Key Components

**Roadside Units (RSUs):** These are special wireless communication devices or base stations mounted along the road to provide connectivity and information support to moving vehicles within their range. RSUs communicate with IoV nodes (OBUs) via message exchanges within their communications zone. They usually have better storage capacity and computation power than the IoV nodes. In this work, we model the RSU as a warrant issuer that permits the IoV node to outsource data to the cloud server. The cloud server only receives data for storage if the IoV node provides a valid warrant from the RSU.
**IoV node (OBU):** These are vehicles deployed with Internet of Things sensors that are able to collect, compute and send data to the cloud server or an edge server. They have inbuilt clocks that are used in timestamping communication messages. They are the main data generators on the network. They are tamper-proof; hence, information stored on them, such as secret key, cannot be uncovered. They communicate with the RSUs to obtain a warrant to outsource the data collected or generated to the cloud or edge server. In our model, they create transactions on the blockchain and store the hash of the corresponding data generated, before outsourcing the data to the cloud or edge server.
**Key Generation Center (KGC):** This is a trusted entity that manages keys used in communication in our network. It registers RSUs and IoV nodes and generates partially private key and pseudo-identities ($PS_{ID}$), for anonymity of entities communicating. It stores a mapping of the assigned pseudo-identity with the actual identity and public key in a hash map in its database. It can trace and revoke the identity of malicious entities. The KGC also has enormous computation resources.
**Cloud or Edge Server:** This is a high-end computational resource and a huge storage-enabled server. Considering the limited resources of the IoV nodes for storage and managing large data generated, the cloud or edge server is the perfect solution. It provides unlimited data processing and storage resources to the IoV nodes. The IoV nodes outsource the data generated to the cloud server.

### 5.3. Layers Design

**Figure 5:**
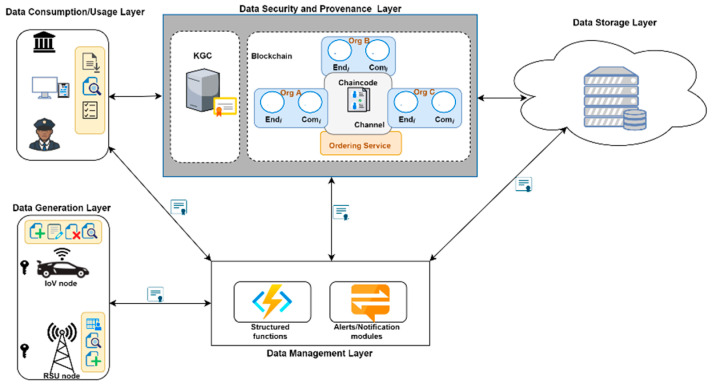
VBlock design with five main layers and individual components.

We grouped the various entities of our model into five layers, namely: data generation layer, data management layer, data security and provenance layer, data storage layer, and data consumption/usage layer. Figure 5 shows the various layer groupings of our model.

**Data Generation Layer:** This layer consists of vehicles deployed with IoT-enabled devices or simply IoV nodes and roadside units (RSUs). The IoV node sends generated or collected data to the data storage layer via the data management layer after it receives a warrant from the RSU. The data includes the GPS location data, speed, and safety condition status. The data cannot be outsourced without receiving a warrant from the RSU node.
**Data Management Layer:** This layer consists of library functions that allow access and process requests received from the data generation layer or consumption layer. It focuses on some specific data processing and operations. Processing of requests in the system includes access to cloud storage data. It also interfaces with the data security and provenance layer. Additionally, it has structured functions that send activities to the data security and provenance. Both the data generation layer and consumption layer interface directly with the data management layer for the processing of requests.
**Data Security and Provenance Layer:** This layer is responsible for data security and auditing. This layer stores hashes of corresponding data needing to be secured from illegal modification. It ensures the immutability of stored data which helps to ensure data provenance. It keeps track of changes made to stored data. It also ensures the security of communication by providing the underlying security communication scheme.
**Data Storage Layer**: This layer is responsible for the scalability of the IoV system applications by providing a distributed or parallel computing environment. This layer plays a major role in storing and managing the IoV data received from the IoV node. Sensors on the vehicles continuously generate a significant amount of data that is collected and managed by the data storage layer.
**Data Consumption/Usage Layer:** The layer consists of all kinds of user classifications, the aim of which are to access the outsourced data from the system for research, investigation, or other useful purposes. Most users at this level help analyze the data received by the cloud server for research purposes. Some of these users include insurance companies, security agencies such as police, healthcare organizations, research institutions such as universities, and governmental bodies.

### 5.4. Communication Design

**Figure 6:**
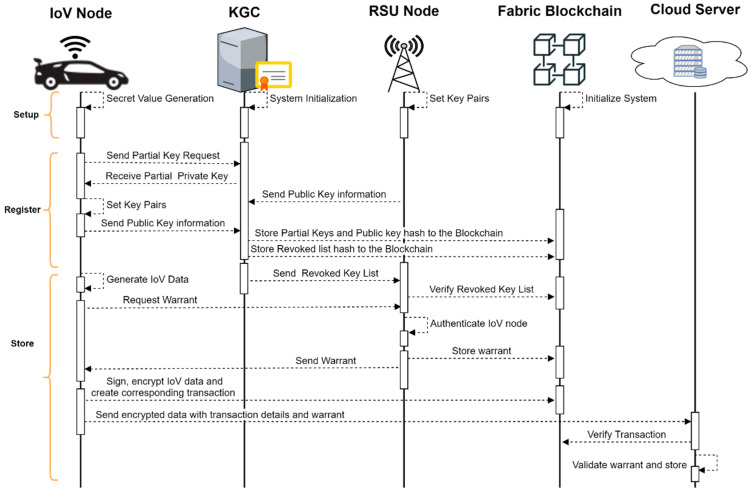
Communication flow between the nodes in the system.

In this section, we present the communication flow of our model to achieve data security and integrity. We outline the communication structures that realize secure data transmission by the nodes and collusion-resistant features of the system. The first stage involves the initialization of the system, where relevant system parameters are set and the blockchain network initiated. The vehicle sends its real identity to the KGC. The KGC verifies the uniqueness of the IoV node’s identity and generates a pseudo-identity $PS_{ID}$ for the IoV node if the real ID submitted is not tagged as revoked. The KGC then generates the partial private key for the IoV node. The KGC sends both $PS_{ID}$ and partial private key to the IoV node. IoV verifies the KGC and generates its full private key and public key. The IoV node sends its public key to the KGC. A transaction is created on the blockchain for the public key, and the pseudo-identity is generated to secure the public key information from any future tamper attacks. For the IoV node to outsource generated data to the cloud, it sends a warrant request to the RSU node. The IoV node signs repeated messages requesting for a warrant to the nearby RSU node. This is done at an interval of 100–300 ms, according to the DSRC protocol. The receiving RSU node verifies the message to ensure that the request is from a legitimate IoV node. The IoV node is also required to send clock information which is used by the RSU to avoid replay attacks. The RSU compares the clock information received to its own to determine the difference. If the difference exceeds the specified threshold, the message is dropped. We further adopt the mechanism described in [40] to increase the security in detecting malicious nodes. To reduce the latency in communication exchanges for every warrant issuing process, we have designed the RSU node to periodically fetch revoked public key lists from the KGC. For every list fetched from the KGC, it is verified from the blockchain using the stored hash values to ensure that it corresponds to the data stored earlier by the KGC on the blockchain. In a situation where the list does not correspond to the hash stored on the blockchain, it means the KGC is compromised, and therefore the list cannot be used [41]. The RSU node responds with the warrant on successful verification of the public key information and pseudo-identity of the IoV node. Finally, the IoV node signs and encrypts the IoV data, creates a corresponding transaction on the blockchain using the steps described in Section 4.4 of this paper, and outsources the data to the cloud server. The cloud server checks the validity of the warrant and transaction. If successful, the cloud server accepts the data. We provide a sequence diagram in Figure 6 that illustrates the processes involved.

### 5.5. Key Revocation

Key revocation comprises the steps taken to remove malicious or compromised IoV nodes from the network. The RSU node is designed to initiate the key revocation process in the system. It uses the message exchange mechanism required to issue a warrant to the IoV node to determine if the IoV node is compromised or malicious. The detailed process is described below.

The RSU node, on receipt of a warrant request, sends a verification request message with a random number r, encrypted with the public key of the IoV node. The IoV node is expected to send a response verification with a random value r+1 within a valid time period that is less than the threshold time interval (THs) of the system. The RSU node, on receipt of the response verification, compares the clock information received to its clock. If the time interval is within the specified threshold value and the random value is equal to r+1, the message exchange continues with the issuance of the warrant. If otherwise, the RSU node logs the IoV node’s information into its database of malicious lists. The RSU queries its database of malicious lists for the current IoV node’s information. If the query result returns false, the current IoV node is marked as malicious, and its information is stored in the RSU’s database. The RSU now bundles the IoV node’s information ($PS_{ID}$, public key $PK_{ID}$, and other information) and sends a revocation request to the KGC. The KGC validates the revocation request and extracts the IoV node’s real information. It marks the IoV node as revoked and creates a corresponding transaction on the blockchain to secure the list of revoked IoV nodes.

### 5.6. Assumptions

Assumptions for the proposed architecture are as follows:

- All communication with the KGC is done via a secure channel.
- IoV nodes connect with the RSUs through a secure channel.
- IoV node uses a secure communication channel to outsource the generated data to the cloud server.
- The IoV nodes and RSUs have a secure communication channel with the blockchain network.

### 5.7. Construction of VBlock

We leverage an efficient certificateless public key cryptography scheme [42] to construct VBlock. We anonymize the real identity of nodes with a pseudo-identity to achieve conditional privacy of the nodes. 

#### 5.7.1. Setup

In this phase, the algorithm sets the parameters and the secret parameters needed to initialize the system. The KGC runs this algorithm with input k and outputs the public parameters params. It determines the pairing parameters: $G_{1}$ and $G_{2}$ of prime order q, with bilinear map $e:G_{1}\times G_{1}\to G_{2}$. It chooses the generator $P\in G_{1}$. It chooses the hash functions: $H_{1}:{\left\{0,1\right\}}^{*}\to Z_{p}^{*}$, $H_{2}:G_{1}\to Z_{p}^{*}$, $H_{3}:G_{2}\to {\left\{0,1\right\}}^{n}$, $H_{4}:G_{2}\to Z_{p}^{*}$, where n is the number of message bits being sent. It randomly chooses $\left(s_{1},s_{2}\right)\in \;Z_{p}^{*}$ a master secret key (MSK), then set $P_{pub}=\left(s_{1}P,\;s_{2}P\;\right)$ as its master public key (MPK). It publishes the public parameters, params = $\left(p,\;q,G_{1}\;,\;G_{2},\;e,\;g,P_{pub},H_{1},\;H_{2},\;H_{3}\right)$ where $g=e\left(P,P\right).$

This phase also includes the creation of the various blockchain accounts for the nodes of the model.

#### 5.7.2. Register

In this phase, the real unique identity $R_{ID}$ of the IoV node is taken to generate the pseudo-identity and partial private key. The IoV node selects a secrete value b, computes $PS_{ID,1}=bP$ and sends $\left(R_{ID}\;,\;PS_{ID,1}\right)$ to the KGC. The KGC generates the pseudo-identity $PS_{ID}$ using the hash function $H_{4}$, which is not published. KGC computes $PS_{ID,2}=R_{ID}⊕\;H_{4}(s_{1}PS_{ID,1}||t\;)\;$ where t is the timestamp. The pseudo-identity is given as $PS_{ID}=\left(PS_{ID,1}\;,\;PS_{ID,2},\;t\right)$. The KGC runs a partial private key generation algorithm with params, msk, and the IoV node pseudo-identity $PS_{ID}\;$ and returns the partial private key $PPK_{ID}$.

KGC sends the pseudo-identity $PS_{ID}$ and partial private key $PPK_{ID}$ to the IoV node via a secure channel. The IoV node uses the partial private key and pseudo-identity to generate its full key pairs, i.e., public and private key. The generated public key is $PK_{ID}$ and the private key is $PSK_{ID}$; the IoV node broadcasts the public key $PK_{ID}$ to the KGC. The KGC creates a transaction on the blockchain for every public key received and its corresponding pseudo-identity.

#### 5.7.3. Store

We describe the mode of outsourcing the data to the cloud server. An RSU node with its identity denoted by $RSU_{ID}$, with secrete key $\alpha_{RS}$ and corresponding public key as $PK_{RS}=\alpha_{RS}P,$ computes a warrant $W_{RS}$ to authorize the IoV node to outsource IoV data to the cloud. The warrant includes a validity period $T_{RS}$ and some auxiliary information $Aux_{RS}$.

(1) $$WA_{RS}=RSU_{ID}\|\;PS_{ID}∥T_{RS}\|Aux_{RS}$$

(2) $$W_{RS}=\alpha_{RS}\cdot H\left(WA_{RS}\right)$$

An IoV node generates data (message) M, receives the warrant, encrypts the data, creates a transaction on the blockchain, and sends the encrypted data together with the transaction ID to the cloud server, CS. By the certificateless encryption in [42] it encrypts M as

(3) $$C=Enc_{CL}\left(M\|WA_{RS}\|W_{RS}\right)$$

With the current time t, the IoV node extracts the hash value of the latest block to be added to the blockchain. We denote this as $VBhash_{t}$.

The IoV node creates a transaction Tx that is endorsed and recorded into the block and sends the transaction details to the cloud server with the data value of the transaction as $VBhash_{t}\|h\left(RSU_{ID}\right)\|h\left(C\|WA_{RS}\|W_{RS}\right)$. 

The IoV node sends $\left(VBhash_{t\;},C\;,WA_{RS}\;,W_{RS}\right)$ and Transaction ID, $Tx_{ID}$ to the cloud server.

The cloud server confirms the transaction from the state transition of the ledger and checks the validity of $T_{RS}$ and $VBhash_{t}$ by the equation:

(4) $$e\left(W_{RS},P\right)=e\left(H\left(WA_{RS}\right),\;PK_{RS}\right)$$

#### 5.7.4. Audit

Given the IoV Data $\left(RSU_{ID\;},C\;,WA_{RS}\;,W_{RS}\;,\;Tx_{ID}\right)$, the auditor is capable of checking the correctness and timeliness via the following:

- Trim the IoV data and obtain $\left(C\;,WA_{RS}\;,W_{RS}\;,\;Tx_{ID}\right).$
- Extract the corresponding transactions from the blockchain.
- Check if the number of transactions created correspond to the number of stored IoV data. If the verification fails, reject.
- Check the validity of $W_{RS}$ and $WA_{RS}\;$. Reject if the validity check fails or is invalid.
- Verify the IoV data timelines by verifying the time of the transaction and reject if the check fails. The transaction time can be obtained from the block.
- Compute $VBhash_{t}\|h\left(RSU_{ID}\right)\|h\left(C\|WA_{RS}\|W_{RS}\right)$ and confirm it is the same as the transaction information.

**Figure 7:**
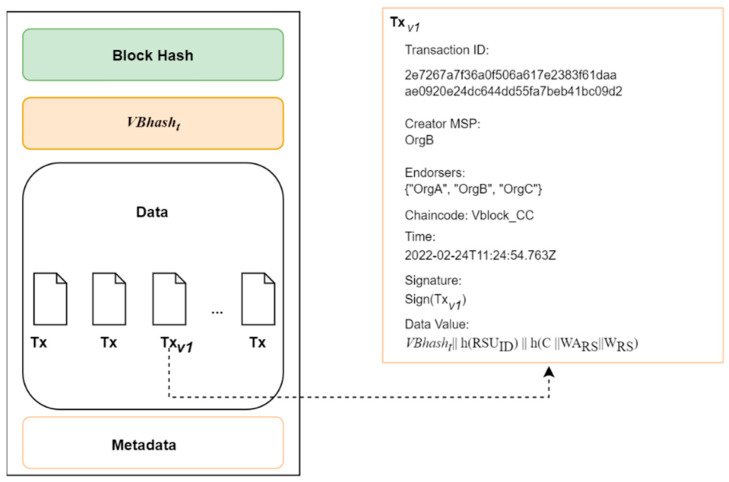
Transaction on the Hyperledger blockchain by the IoV node for outsourcing data.

If the verifications above are successful, the timeliness and correctness of the IoV data is guaranteed. Figure 7 shows a transaction on the blockchain for outsourcing data.

### 5.8. Algorithms

We provide the three main algorithms needed to achieve our proposed model for outsourcing data to the cloud server. The description is as follow.

We denote endorsing peers as $End_{i}$, public key and private key of endorsing peers as $End_{PKi}$ and $End_{SKi}$ respectively. We denote committing peers as $Com_{i}$, public key and private key of committing peers as $Com_{PKi}$ and $Com_{SKi}$ respectively. We denote chaincode as $C_{HC}$, numbers of endorsing peers as ‘l’ and number of committing peers as ‘m’. The endorsement policy is represented as EP and Transaction ID as $Tx_{ID}$. We show the algorithm for requesting warrant from RSU node in Algorithm 1. We also show the algorithm for creating a transaction on the blockchain for data generated in Algorithm 2. We finally show the algorithm for outsourcing data to the cloud server in Algorithm 3.
**Algorithm 1 Requesting for Warrant from RSU node****Require:** $IoV_{ID}$, $PK_{ID}$, $PS_{ID}$, $PSK_{ID}$, $RSU_{ID}$, **1:** $IoV_{ID}$ signs warrant request message $MR_{wi}$: $W_{Ri}$ = sign ($MR_{wi}$,$\;PS_{ID}$, $PSK_{ID}$) **2:** $IoV_{ID}$ sends $W_{Ri}$ to nearest $RSU_{ID}$. **3:** $RSU_{ID}$ checks **4:**   Condition 1: Verify ($PK_{ID}$, $PS_{ID}$, $MR_{wi}$,$\;W_{Ri}$) **5:**   Condition 2: Check if $PS_{ID}$ or $PK_{ID}$ is not revoked **6: if** (Condition 1 && Condition 2) = True **7:**   $RSU_{ID}$ computes warrant **8:**            $WA_{RS}=RSU_{ID}\;PS_{ID}∥T_{RS}Aux_{RS}$ **9:**            $W_{RS}=\alpha_{RS}\cdot H\left(WA_{RS}\right)$ **10:**   $RSU_{ID}$ sends warrant $(W_{RS}$, $WA_{RS})$ to $IoV_{ID}$ **11: else** return fail **end if**
**Algorithm 2: Creating Transaction for Data Generated****Require:** $IoV_{ID}$, $PK_{ID}$, $PS_{ID}$, $PSK_{ID}$, M, $End_{i}$, $Com_{i}$**1:** $IoV_{ID}$ computes ciphertext C of the data generated M **2:** $IoV_{ID}$ extracts $VBhash_{t}$
**3:** $IoV_{ID}$ computes $VBhash_{t}h\left(RSU_{ID}\right)h\left(CWA_{RS}W_{RS}\right)$ **4:** $IoV_{ID}$ sets transaction proposal Tx = $VBhash_{t}h\left(RSU_{ID}\right)h\left(CWA_{RS}W_{RS}\right)$ **5:** $IoV_{ID}$ signs Transaction Proposal Tx: STx = sign (Tx,$\;PS_{ID}$, $PSK_{ID}$) **6:** $IoV_{ID}$ sends STx to endorsers $End_{i}$, **7: for** $End_{i}\left(1\leq i\leq l\right)$ check **8:**   Condition 1: Verify ($PK_{ID}$, Tx, STx) **9:**   Condition 2: Execute chaincode $C_{HC}$ and check format of Tx
**10:**
**if** (Condition 1 && Condition 2) = True **11:**   $End_{i}$ signs STx: ${\mathrm{STx}}^{\prime }$ = sign (STx, $End_{SKi}$) **12:**    Send transaction proposal response ${\mathrm{STx}}^{\prime }$ to $IoV_{ID}$ **13: else** return fail **end if** **14: end for** **15:** $IoV_{ID}$ sends ${\mathrm{STx}}^{\prime }$ to ordering service and wait for acknowledgment **16: for** $Com_{i}\left(1\leq i\leq m\right)$ check **17:**   Condition 1: Verify ($End_{PKi}$, $PK_{ID}$, STx, ${\mathrm{STx}}^{\prime }$) **18:**   Condition 2: Check Endorsement Policy EP **19:**
**if** (Condition 1 && Condition 2) = True **20:**   Set the transaction status = valid **21:**   Validate consensus and add transaction to block **22:**   Send acknowledgment $Tx_{ID}$ to $IoV_{ID}$ **23: else** **24:**   Set transaction status = invalid; **25:**    Send acknowledgment to $IoV_{ID}$ **26: end if** **27: end for**


**Algorithm 3: Outsourcing Data to the Cloud Server**
**Require:** $IoV_{ID}$, $PK_{ID}$, $PS_{ID}$, $PSK_{ID}$, CS, $C\;,\;\;WA_{RS},\;\;W_{RS},\;\;Tx_{ID}$ **1:** $IoV_{ID}$ signs and sends $\left(VBhash_{t\;},C,WA_{RS},W_{RS},\;\;Tx_{ID}\right)$ to cloud server CS **2:** CS checks **3:**   Condition 1: Verify ($PK_{ID}$, $sign\left(VBhash_{t\;},C,WA_{RS}\;,W_{RS}\;,\;\;Tx_{ID}\right)$) **4:**   Condition 2: Verify transaction $Tx_{ID}$ validity **5:**   Condition 3: Verify warrant; compute $e\left(W_{RS},P\right)=e\left(H\left(WA_{RS}\right),\;PK_{RS}\right)$ **6: if** (Condition 1 && Condition 2 && Condition 3) = True **7:**   CS, accept and store data $\;\left(C,\;\;WA_{RS},\;\;W_{RS\;,\;\;\;}Tx_{ID}\right)$ **8: else** return fail **end if**

## 6. Security Analysis

### 6.1. Security against Forgery and Modification Attacks 

VBlock is secure against forgery attacks conducted by any adversary. It is not possible for an adversary to modify a transaction in the blockchain for corresponding data, even if the adversary forges IoV data. If a semi-trusted IoV node (user) tries to forge IoV data, it may conduct the following attacks: 1. The IoV node will outsource its generated data to the cloud server but attempts to convince the cloud server that the IoV data were generated by another IoV node. 2. The IoV node tries to replace or modify existing IoV data with new data by colluding with the cloud server.

For attack 1, in order to validate the data being outsourced by the IoV node, we require the RSU nodes to generate a warrant to the IoV node to allow the IoV node to outsource the data. The warrant includes the RSU node identity, IoV node pseudo-identity, validity period, and other additional information recorded on the blockchain. The warrant is unforgeable as it is constructed based on a secure signature scheme [43]. Hence it is computationally impossible for the IoV node to conduct attack 1. For attack 2, the IoV data generated by the IoV node is recorded as a transaction on the blockchain. If a node tries to replace stored IoV data with new ones, the only feasible way is to fork the blockchain and cause the majority of consensus nodes to accept the blockchain with the corresponding transaction of the new IoV data generated. At this point, the security against forgery attacks is based on the fundamental blockchain security. Even if there is collusion among the three entities, the IoV node, a malicious RSU node and the cloud server, the IoV node cannot succeed in conducting attack 2.

### 6.2. VBlock Guarantees the Timeliness of IoV Data

The timeline for every outsourced data corresponds to a transaction time on the blockchain. In VBlock, each datum maps to one transaction in the blockchain. This makes it possible to efficiently retrieve the time when the IoV data was generated and outsourced to the cloud server. Hence there is a guarantee of timeliness of IoV data.

### 6.3. VBlock Guarantees Public Key Security

Since we integrate the public key information into the blockchain as transactions, this makes it impossible for an adversary to tamper with the user’s public key considering the current computing power and the adversary’s attack ability. Even if an attacker succeeds in breaking the security of the KGC, the attacker cannot break that of the blockchain. In summary, the computing power required for an attacker to decipher the VBlock’s model exceeds the attacker’s deciphering ability. Hence the public key in the system is safe.

### 6.4. Necessity of Blockchain Integration

In the absence of blockchain, VBlock is unprotected from data forgery, modification, and deletion attacks without noticing when there is collusion. Moreover, as the load on the central KGC increases extremely, it may cause the system to fail. When the central server or KGC is tampered with, it may cause huge problems for the system. However, in VBlock, IoV data generated by the IoV node and the activities performed by the KGC and RSU are all integrated into the blockchain as transactions. This forms the basic principle of VBlock. Hence, provided the tamper-resistant nature of the blockchain is guaranteed, the correctness, security and integrity of outsourced IoV data in VBlock is also guaranteed. This makes the blockchain technology a key necessity in VBlock.

### 6.5. VBlock Is Resistant to Replay Attacks

A replay attack is the repeated process of transmitting valid data packets or messages that have already been used in previous communications by a malicious mode. Attackers use this mechanism to deceive the receiver of the packet or message into believing that the malicious node is a legitimate node. This model uses the clock difference (threshold range) and random number challenge to mitigate replay attacks on the network. Only messages within the threshold range and with the right response to the random number challenge are considered valid, and hence resistant to replay attacks.

### 6.6. VBlock Ensures Data Access Control

VBlock access control intends to constrain the type of resources or data that members of the network are authorized to see. The intended data security and privacy level setting is achieved through channel configuration provided by the Hyperledger platform. Hyperledger Fabric allows adjustment of data transparency levels to any desired use case via channels setup or private data specification.

## 7. Performance Evaluation

We present the analysis and performance efficiency of our model with respect to computation costs and simulation throughputs in this section. We also discuss the simulation details of our proposed system.

### 7.1. Evaluation Metrics

We analyze the computational cost involved in our model and the basic metrics of the Hyperledger Fabric setup, which include success rate, transaction latency, and transaction throughput. We briefly explain these below.

- The number of successful transactions executed out of the total transaction is known as the success rate.
- Latency refers to the time interval between the transaction initialization and the actual transaction execution.
- Throughput is the number of successful transactions per second.

### 7.2. Computation Cost

The execution time of the cryptographic operation was computed using the experiment in [42] with the Pairing Base Cryptography (PBC) Library. We used a computer with a Linux environment running the Ubuntu 20.04.4 LTS operating system with Intel(R) Core (TM) i9-10900K CPU @ 3.70GHz @3.70 GHz and 32GB RAM. We utilized bilinear pairing $e:G_{1}\times G_{1}\to G_{2}$, a Type A pairing constructed from the elliptic curve $y^{2}=x^{3}+x$ over a finite field with 128 bits of security levels. Table 1 presents the running times and symbols for the various operations. Table 2 shows the total computational cost in message exchanges and outsourcing data to the cloud server in VBlock.

We further provide a computation cost comparison of our model with three other high-impact published works that are most related and have the same entities as ours. Table 3 shows the comparison of computation costs by the various entities in message exchanges and data storage processes. Only our model and Ali et al. provide information on the data storage process for the network. Our model achieves lower costs than some other models. Although our model takes slightly more time than some of the other models, it achieves all security attributes elaborated in this work. This is the trade-off we get in achieving a complete collusion-resistant and tamper-proof model. 

### 7.3. Simulation

We simulated our model on Hyperledger Fabric with a network consisting of six peers, owned and contributed to by three organizations. All the peers on the network run as containers. All the containers run on an independent physical device on a Local Area Network (LAN), with each physical device running the Ubuntu 20.04.4 LTS operating system with Intel(R) Core (TM) i7-7700 CPU @3.60 GHz @3.60 GHz with 8GB RAM and Fabric V2.0 installed. A 1000Mbps Ethernet switch was used to connect all physical devices. We use GO to implement our Fabric chaincode and Node.js for the Hyperledger Fabric Client SDK. We use a crash fault-tolerant (CFT) ordering service called raft [44] to achieve consensus in transaction ordering on the network. We used three peers as endorsers and three peers as committers of the network. The conceptual representation of the implemented model is shown in Figure 3. We installed and instantiated the chaincode on the endorser peers. We used the default “N of N” policy as our endorsement policy i.e., all three organizations are required to endorse a transaction to make it valid. To measure the performance of our blockchain system, we used Hyperledger Caliper [45]. This is a blockchain benchmark tool that allows performance measurement on blockchain implementations. We present the simulation and Hyperledger Caliper setup environment in Table 4.

We designed two experiments to evaluate the performance of our model. We used five sets of node categories ranging from 100, 200, 300, 400 and 500 nodes to query the system and at the same time to make comparisons of the various experiments designed. Each experiment category was tested five times to ascertain the average of the experimental results.

- Experiment 1 was designed to evaluate the transaction per second (TPS) and latency of open functions (create, update, or delete) of the network. This is to measure the process of outsourcing data to the cloud server.
- Experiment 2 was designed to evaluate the transaction per second (TPS) and latency of query function of the network. This is to measure the performance of verifying information from the blockchain network.

### 7.4. Results and Discussion

**Figure 8:**
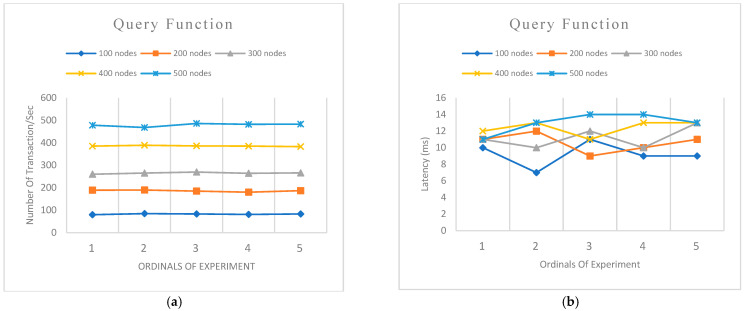
System (**a**) Transactions per second (TPS) of query function with varying node groups (**b**) Latency of query function with varying node groups.

From Figure 8a, it can be seen that an average of 82 transactions per second (TPS) for the node category with 100 nodes was recorded for the query function. The TPS consistently increased, nearing the number of nodes querying the system. The latency recorded in Figure 8b for nodes ranging from 100–400 stabilized around 12 ms. A slight increase in latency is observed when the nodes increase to 500.

**Figure 9:**
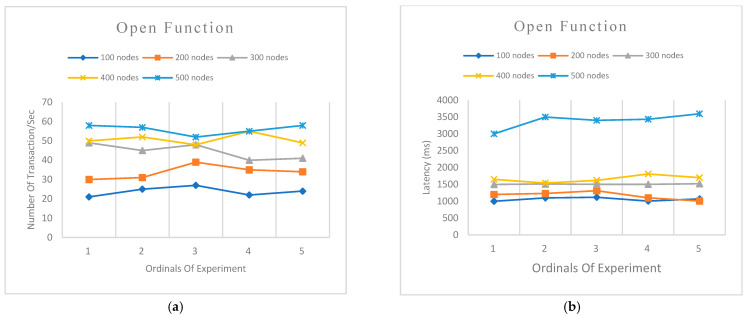
System (**a**) Transactions per second (TPS) of open function with varying node groups (**b**) Latency of open function with varying node groups.

Figure 9a shows the evaluation of transactions per second (TPS) for open functions. An average of 23 TPS was recorded for the 100 nodes category, 33 TPS for 200 nodes, 44 TPS for 300, 50 for 400 nodes, and 56 for 500 nodes. The TPS consistently increased as the number of nodes requesting simultaneously also increased.

Success rate: We obtain a 100% success on both open and query functions with simultaneous transactions on the same number of nodes categories.

Throughput: We observe that query function throughputs are higher than the open function. Although both functions record an increase in throughput as the number of transactions increases, they both show slight consistency in their throughputs. The consistency in throughputs recorded demonstrates the availability and reliability of the Hyperledger network.

**Figure 10:**
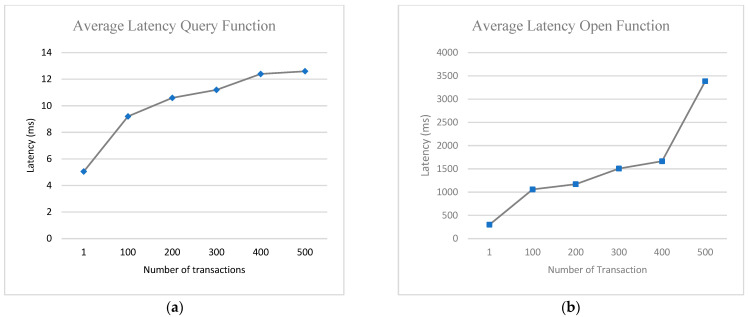
System average latency for (**a**) Query function (**b**) Open function with varying number of transactions.

Latency: Figure 10 shows the average latencies of query functions and open functions. The open function processes are generally higher than that of query functions. This is due to the additional time needed to complete the endorsement, ordering and validation. The query function needs between 5 milliseconds to almost 13 milliseconds to complete the function whenever the server receives 500 requests. However, the open function needs more time because it requires the addition of a new block to the ledger. The open function needs between 300 milliseconds to 3500 milliseconds when the server receives 500 requests. The general latency of both query and open function increase with a corresponding increase in the number of nodes requesting at the same. It is worth noting that test patterns being developed to improve smart contract execution time will reduce blockchain computation costs [46].

**Figure 11:**
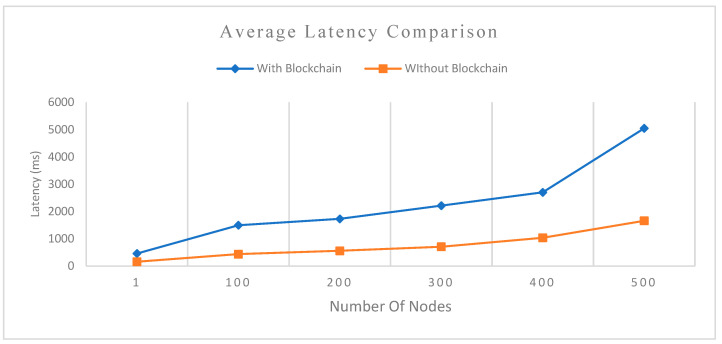
Average latency of VBlock data outsourcing model with and without the blockchain.

In Figure 11, a very important observation recorded in the latencies of VBlock with the blockchain is the considerable increase in latency for outsourcing data to the cloud server, with a simultaneous increase in the number of nodes. This is a result of the trade-off between attaining high security, tamper-proofing, and data provenance over low latency.

Table 5 shows the comparison of our model to other existing models and the literature discussed in this paper. A careful analysis of the various metrics used indicates that VBlock shows higher advantages over other systems proposed. 

## 8. Conclusions

In this paper, we have presented VBlock, a secure data outsourcing model for IoV networks that leverages the Blockchain to keep immutable records. The design utilizes blockchain to effectively secure outsourced data from illegal modifications and ensures data provenance and auditing. We introduced a key revocation mechanism to further secure the IoV network from malicious or compromised nodes. We analyze the performance of VBlock while comparing it to other proposed methods of IoV systems, as well as comparing it with current cutting-edge solutions to data outsourcing to cloud service providers. The security of VBlock can be guaranteed even if there is a collusion between the creator of the outsourced data and the cloud server. VBlock is built on a Hyperledger Fabric blockchain where access to the network is limited to only known nodes with increased security and privacy. The correctness and security are also dependent on the security of the Hyperledger Blockchain. The performance efficiency from our experimental results shows good throughputs with low latencies, which makes this model practicable. By implementing the proposed model, the future generation of a safe smart city can be achieved such that outsourced data by the IoV network can be fully trusted for usage in smart city management and improvement. We envision expanding the data availability from IoV networks in our future works.

## Figures and Tables

**Table 1:** Cryptographic operations running times.

Description	Symbol	Running Time (ms)
Encrypt message by certificateless encryption	$T_{enc}$	2.3615
Exponentiation in $G_{2}$	$T_{ex2}$	0.987
Bilinear pairing operation	$T_{bp}$	3.038
Hash function	$T_{h}$	0.0003
Point multiplication	$T_{m}$	0.0193
Point addition	$T_{pa}$	0.081
Open Function Transaction on Blockchain	$T_{xO}$	304.42
Query Function Transaction on Blockchain	$T_{xQ}$	5.005

**Table 2:** Computational Cost Estimation.

Nodes	Message Exchanges	Data Outsourcing/Storage
RSU	$2T_{m}$$+2T_{h}$$+T_{bp}$$+T_{xQ}$ ≈ 14.5056 ms	$T_{h}$$+T_{xO}$ ≈ 304.4203 ms
IoV node	$2T_{m}$$+3T_{h}$$+T_{bp}$ ≈ 3.4249 ms	$3T_{h}$$+2T_{m}$$+T_{enc}$$+T_{xO}$ ≈ 306.821 ms
Cloud Server	$2T_{m}$$+4T_{h}$$+2T_{bp}\;$$+T_{xQ}$ ≈ 11.4682 ms	None
KGC	$2T_{m}$$+2T_{h}$$+T_{bp}$ ≈ 3.4246 ms	$T_{h}$$+T_{xO}$ ≈ 304.4203 ms

**Table 3:** Computational Cost Comparison.

Models	Nodes	Message Exchanges	Data Outsourcing/Storage
[15]	RSU	$2T_{m}$$+2T_{h}$$+3T_{pa}$ ≈ 0.2822 ms	−
	IoV node	$T_{m}$$+T_{h}$$+T_{pa}$ ≈ 0.1006 ms	−
	KGC	$T_{m}$$+T_{h}$$+T_{pa}$ ≈ 0.1006 ms	−
[16]	RSU	$2T_{ex2}$$+4T_{h}$ ≈ 1.986 ms	$T_{h}$$+T_{xO}$ ≈ 304.4203 ms
	IoV node	$2T_{ex2}$$+4T_{h}$ ≈ 1.986 ms	−
	KGC/CA	$2T_{ex2}$$+4T_{h}$$+T_{xQ}$ ≈ 6.9802 ms	$T_{h}$$+T_{xO}$ ≈ 304.4203 ms
[21]	RSU	$T_{m}$$+2T_{h}$$+T_{bp}$$+2T_{xQ}$ ≈ 13.0679 ms	−
	IoV node	$2T_{m}$$+3T_{h}$$+T_{bp}$ ≈ 3.4249 ms	−
	KGC	$T_{m}$$+2T_{h}$$+T_{bp}$$+2T_{xQ}$ ≈ 13.0679 ms	−
Ours	RSU	$2T_{m}$$+2T_{h}$$+T_{bp}$$+T_{xQ}$ ≈ 14.5056 ms	$T_{h}$$+T_{xO}$ ≈ 304.4203 ms
	IoV node	$2T_{m}$$+3T_{h}$$+T_{bp}$ ≈ 3.4249 ms	$3T_{h}$$+2T_{m}$$+T_{enc}$$+T_{xO}$ ≈ 306.821 ms
	KGC	$2T_{m}$$+2T_{h}$$+T_{bp}$ ≈ 3.4246 ms	$T_{h}$$+T_{xO}$ ≈ 304.4203 ms

**Table 4:** Simulation and Hyperledger Caliper environment setup.

Component	Description
CPU	Intel(R) Core (TM) i9-10900K CPU @ 3.70GHz 3.70 GHz
Memory	32 GB
Operating System	Ubuntu 20.04.4 LTS
Node.js	v14 LTS
Docker	Version 20.10.11
CLI Tool	Node-gyp
Fabric	V2.2

**Table 5:** Comparison of our model with other blockchain-based related existing systems.

Models	Metrics								
	Blockchain Based	Access Control	Replay Attack Resistance	Tamper-Proof Audit	Forgery Attacks Resistance	Certificateless PKI	Key Revocation Mechanism	Collusion-Resisting Attack	Warrant-Based Data Outsourcing
[15]	NO	YES	YES	NO	NO	YES	YES	NO	NO
[16]	YES	NO	YES	NO	YES	NO	YES	YES	NO
[17]	YES	YES	NO	NO	YES	NO	NO	YES	NO
[18]	YES	YES	NO	NO	YES	NO	NO	YES	NO
[19]	YES	NO	YES	YES	YES	NO	YES	NO	NO
[20]	YES	NO	YES	YES	YES	NO	YES	YES	NO
[21]	YES	YES	YES	NO	NO	YES	YES	NO	NO
[22]	YES	NO	YES	YES	YES	NO	YES	YES	NO
[23]	YES	YES	YES	YES	NO	NO	YES	NO	NO
[24]	YES	NO	NO	YES	YES	NO	NO	YES	NO
**Ours**	**YES**	**YES**	**YES**	**YES**	**YES**	**YES**	**YES**	**YES**	**YES**

## Data Availability

Not applicable.
